# Colonic gallstone ileus treated by a transanal ileus tube followed by spontaneous gallstone dislodgement: A case report

**DOI:** 10.1002/deo2.145

**Published:** 2022-07-08

**Authors:** Tadataka Takagi, Shoichi Kinoshita, Chihiro Kawaguchi, Kuniyuki Kojima, Hirotsugu Ueno, Naoki Nishimura, Naotaka Shimozato, Yasuyo Shirai, Ryuichi Noguchi, Takao Ohyama

**Affiliations:** ^1^ Department of Surgery Heisei Memorial Hospital Nara Japan; ^2^ Gut and Liver Center Heisei Memorial Hospital Nara Japan

**Keywords:** colon, colonoscopy, fistula, gallstones, ileus

## Abstract

A 71‐year‐old obese woman was referred to our hospital with lower left abdominal pain. Computed tomography showed a 46 mm elliptic calcification lodged in the sigmoid‐descending colon junction (SDJ), which had been detected 5 years prior but was not within the gall bladder at presentation. Therefore, we diagnosed colonic gallstone ileus with obstructive colitis caused by a gallstone. Colonoscopy revealed a smooth gallstone impacted at the sigmoid‐descending colon junction, which was not fixed and could be pushed proximally with the endoscope. Dislodgement of the stone was unsuccessful with both a large polypectomy snare and a retrieval basket. Considering the high risk of surgery, we chose a non‐surgical treatment strategy for obstructive colitis. Accordingly, a transanal ileus tube was placed to drain the proximal portion of the gallstone. The drainage of the colon by the ileus tube was satisfactory; the proximal colon was decompressed, ameliorating the obstructive colitis. Five days after tube placement, a colonoscopy revealed spontaneous passage of the gallstone into the rectum where it was finally removed. Cholecystocolonic fistula formation was confirmed by magnetic resonance imaging. We decided to surgically close the cholecystocolonic fistula to prevent future retrograde biliary infections. The surgery used a surgical stapler and was successful, with an uneventful postoperative course. Since radical surgical treatment of colonic gallstones and cholecystoenteric fistulas has a risk of postoperative morbidity and mortality, this case illustrates the importance of thoroughly considering nonsurgical interventions and surgeries for the safe treatment of colonic gallstone ileus.

## INTRODUCTION

Gallstone ileus is caused by intraluminal intestinal occlusion by a biliary calculus and accounts for only 1–4% of all intestinal obstructions.^1^ Nearly 80% of gallstone obstructions occur in the small bowel even though obstructions can occur anywhere between the stomach and rectum.^2^ Colonic ileus is an extremely rare form of gallstone ileus, resulting from gallstones passing into the colon through cholecystocolonic or choledochocolonic fistulas.^1^, ^3^ Although most reported colonic ileus cases have been treated surgically, the postoperative morbidity and mortality rates are high.^1^, ^3^, ^4^ Herein, we report a case of colonic gallstone ileus, which was treated with a transanal ileus tube, causing spontaneous dislodgement of the gallstone.

## CASE REPORT

**FIGURE 1:**
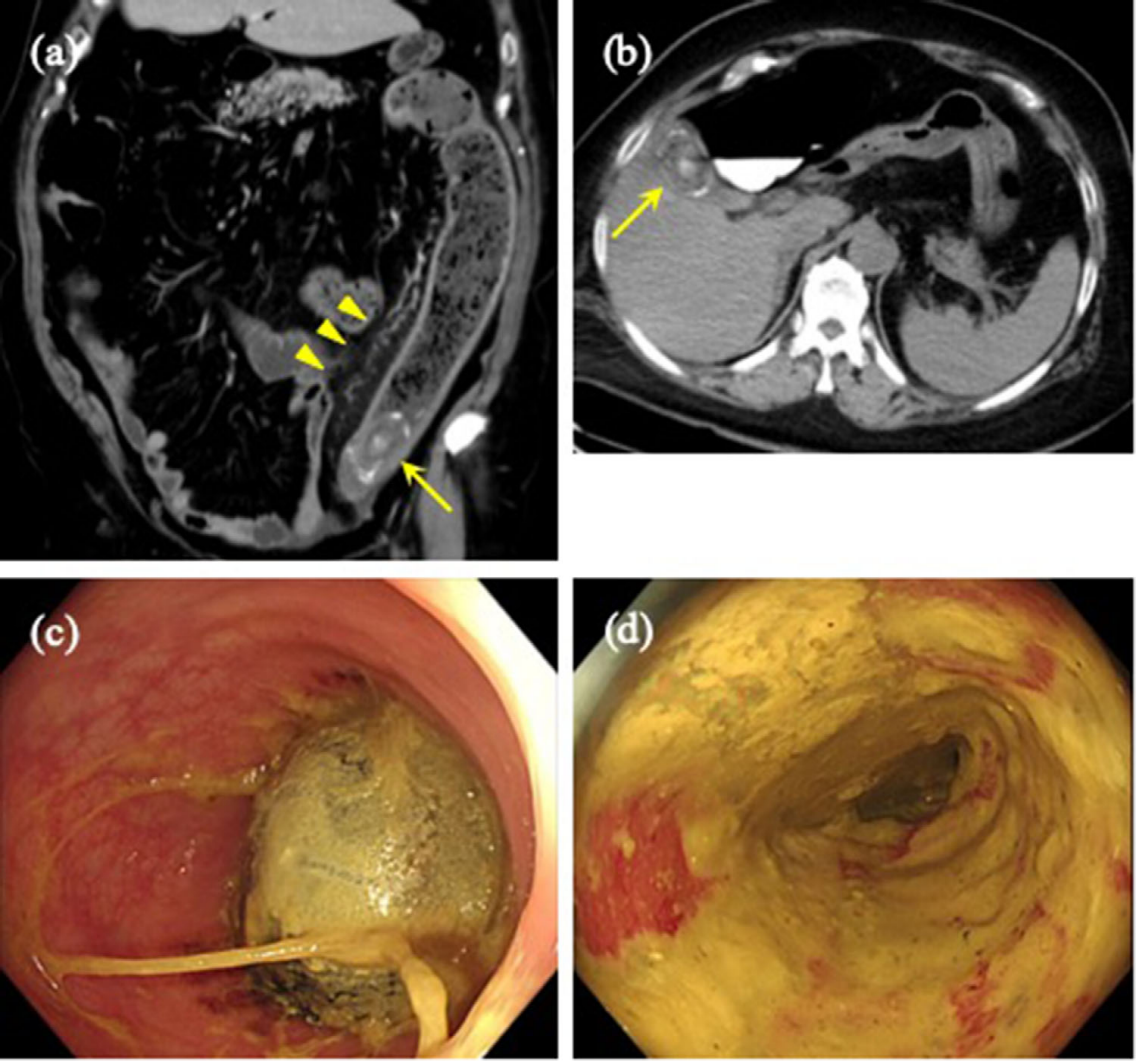
Colonic gallstone ileus. (a) Computed tomography showing a 46 × 30 mm elliptical calcification (yellow arrow) lodged in the sigmoid‐descending colon junction, and dilation of the oral colon with pericolic inflammation (yellow arrowhead). (b) A gallstone of the same measurements was confirmed in a computed tomography scan 5 years prior (yellow arrow) (c) Colonoscopy showing the gallstone impacted at the sigmoid‐descending colon junction. (d) The mucosa proximal to the obstruction appears red and edematous, compatible with obstructive colitis

A 71‐year‐old woman was referred to our hospital for lower left abdominal pain which began 11 days prior to the medical presentation. The patient was obese, with a body composition of 156 cm and 91.5 kg (body mass index = 37.6 kg/m^2^). On physical examination, we observed lower left abdominal pain and tenderness, but no muscular defense. Hematologic tests showed elevated levels of C‐reactive protein (11.4 mg/dl), white blood cell count (15,100/μl), creatinine (1.0 mg/dl), and blood urea nitrogen (26.1 mg/dl). Computed tomography (CT) showed a 46 × 30 mm elliptic calcification lodged in the sigmoid‐descending colon junction (SDJ). The colon proximal to the obstruction was dilated; the bowel walls were thickened, with an elevated density of the surrounding adipose tissue (Figure 1a). There were no areas of poor‐contrast media perfusion, free intra‐abdominal air, or ascites. A gallstone of the same size had been confirmed through CT scans 5 years previously but was absent from the gall bladder in the present examination (Figure 1b). The final diagnosis was a colonic gallstone ileus with obstructive colitis due to an impacted gallstone in the SDJ.

**FIGURE 2:**
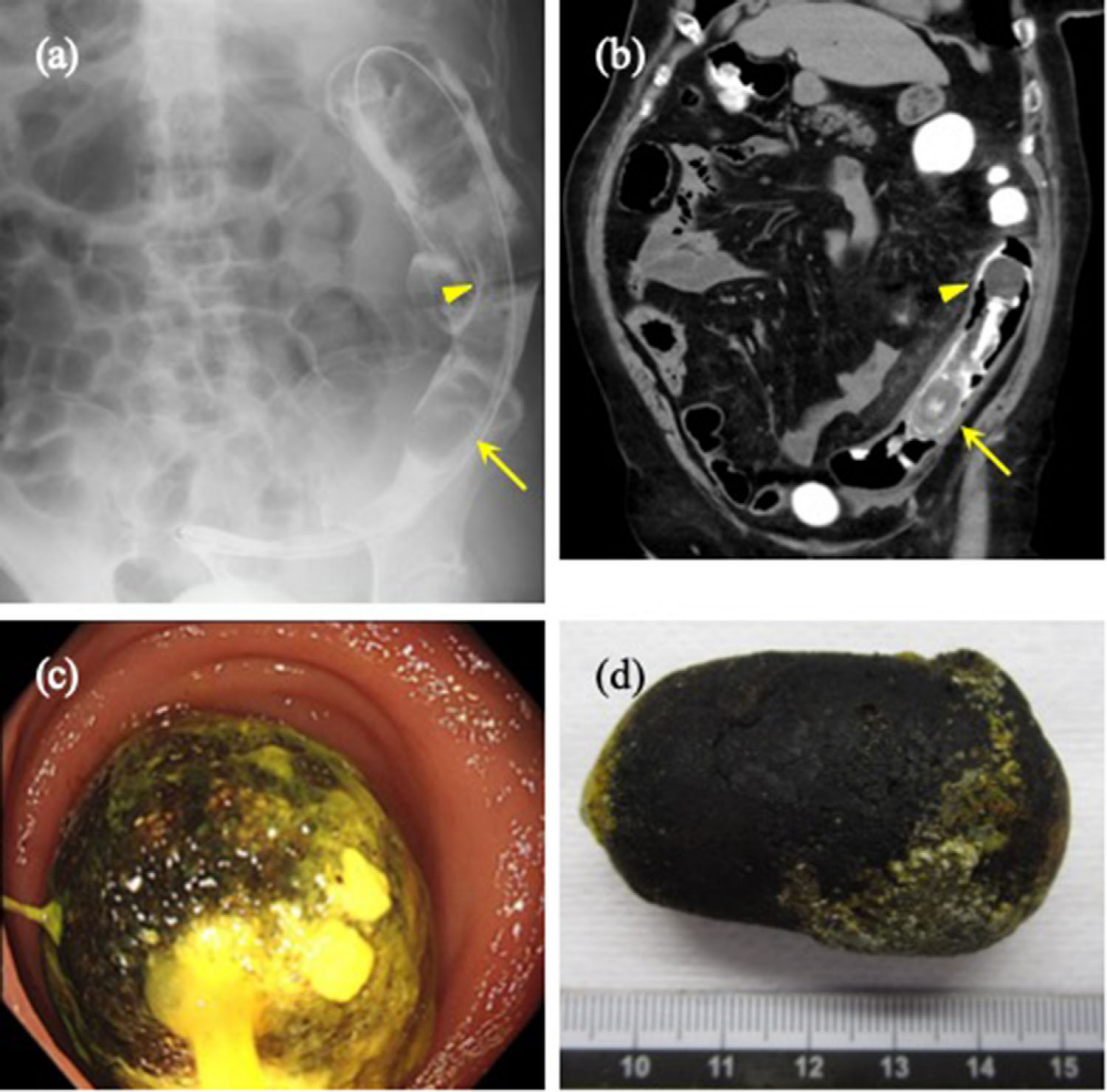
The placement of a transanal ileus tube. The gallstone (yellow arrow) and inflated balloon of the transanal ileus tube (yellow arrowhead) as seen via (a) radiography and (b) computed tomography. (c) The gallstone passed into the rectum 5 days after transanal tube placement. (d) The spontaneously dislodged and evacuated gallstone

A colonoscopy showed a smooth oval gallstone impacted at the SDJ, as was seen in the CT scan (Figure 1c). However, the gallstone was not fixed and could be pushed proximally using the endoscope. The mucosa proximal to the obstruction was reddened and edematous, compatible with the diagnosis of obstructive colitis; however, there was no compression ulcer due to the gallstone (Figure 1d). A large polypectomy snare and a retrieval basket were unsuccessful in dislodging the stone because of the difficulty in grasping its smooth surface. With no clinical symptoms suggestive of impending colon perforation, we decided on a non‐surgical approach to treating obstructive colitis. A transanal ileus tube was placed to drain the proximal side of the gallstone obstruction (Figures 2a,b). Drainage of the colon by the ileus tube was good and the proximal colon was decompressed, followed by an expanded diameter and improved elasticity. Two days after tube placement, the patient's symptoms, laboratory data, and radiological examinations for obstructive colitis improved. However, the gallstone had not been dislodged. Five days after tube placement, a colonoscopy revealed that the gallstone had spontaneously passed into the rectum (Figures 2c,d). Accordingly, the stone was removed. The clinical course was uneventful, and the patient was discharged 9 days after tube placement.

**FIGURE 3:**
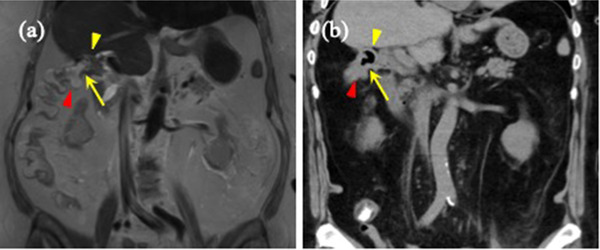
Cholecystocolonic fistula. (a) Magnetic resonance imaging shows the cholecystcolonic fistula (yellow arrow) between the gallbladder (yellow arrowhead) and transverse colon (red arrowhead). (b) Computed tomography showing air in the gallbladder (yellow arrowhead). The positions of the cholecystcolonic fistula (yellow arrow) and transverse colon (red arrowhead) were consistent with magnetic resonance imaging

**FIGURE 4:**
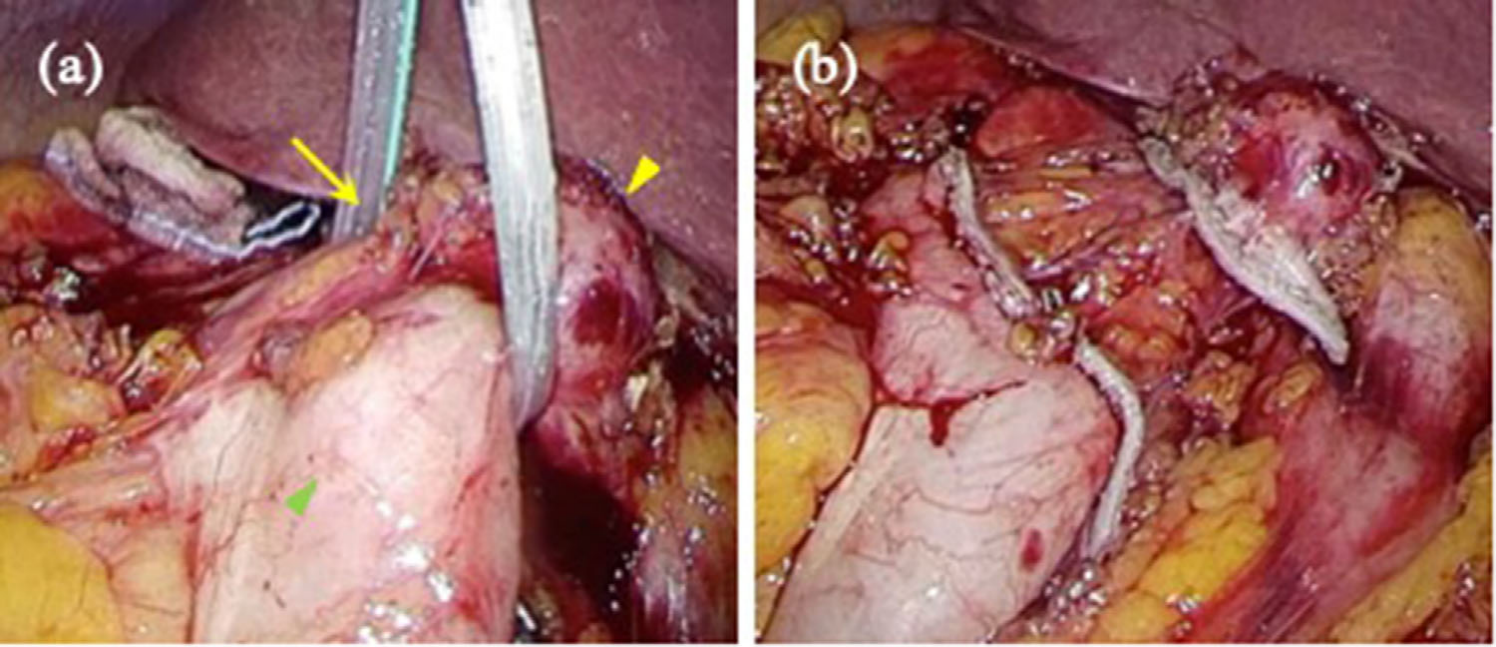
Intraoperative findings. (a) Encircled cholecystcolonic fistula (yellow arrow) between the gallbladder (yellow arrowhead) and transverse colon (green arrowhead). (b) Fistula closure was performed using a surgical stapler

Cholecystocolonic fistula formation was confirmed through magnetic resonance imaging (Figure 3a). Esophagogastroduodenoscopy showed no cholecystoduodenal fistula formation. CT scans showed gas in the gallbladder and common bile duct, indicating communication between the biliary tract and colon (Figure 3b). In addition, no neoplastic gallbladder lesions were observed. Therefore, we decided to surgically close the cholecystocolonic fistula to prevent future retrograde biliary infections. Upon elective laparoscopic surgery, the cholecystocolonic fistula was successfully encircled (Figure 4a), resected from the colon, and closed using a surgical stapler device (Figure 4b). The postoperative course was uneventful, and the patient was discharged 8 days postoperatively.

## DISCUSSION

Gallstone ileus generally requires surgery to remove the gallstone and close the cholecystoenteric fistula.^5^ Surgical methods include extraction by enterotomy or bowel resection. In addition, concomitant fistula closure with or without a cholecystectomy may be performed. However, these methods are associated with a relatively high risk of postoperative morbidities. Furthermore, especially in cases involving a cholecystectomy, high rates of postoperative mortality (7%–33%) have been reported.^1^, ^3^, ^4^, ^5^ Therefore, careful consideration must be made regarding the procedures to perform. Moreover, alternative nonsurgical treatments may be considered depending on the circumstances.

Previous reports of non‐surgical treatment included endoscopic lithotripsy and endoscopic extraction of the colonic gallstone with a snare catheter or retrieval basket.^6^, ^7^, ^8^ In the present case, endoscopic lithotripsy was not performed because the gallstone was too large to fit inside a crusher catheter. Furthermore, endoscopic snare retrieval was unsuccessful because of the smoothness and slipperiness of the gallstone. There have been a few reports of spontaneous resolution of gallstone ileus, with stones passing through the rectum.^9^ However, to the best of our knowledge, this is the first report of spontaneous dislodgement of an impacted colonic gallstone after decompression of the obstructed colon by transanal ileus tube treatment. The ileus tube treatment may have helped in reducing inflammation and restoring the elasticity of the colon walls so that the stone could pass through the SDJ.

Regarding surgical extraction of the colonic gallstone, we hesitated to perform primary surgical treatment not only because colon perforation was not imminent, but also because the patient was morbidly obese. In previous reports of cholecystoenteric fistulas, many patients were obese,^10^ similar to this case. Obesity was presumably associated with the high postoperative morbidity rate reported in those studies. Therefore, an enterotomy was expected to be difficult in this case, considering the massive visceral fat and colonic inflammation. We inserted a transanal ileus tube to decrease the inflammation of the colon. Finally, the gallstone was spontaneously dislodged and evacuated.

For cholecystocolonic fistula closure, we resected the fistula with a surgical stapler but did not perform a colectomy or cholecystectomy. The colon wall at the fistula site was relatively soft; a surgical stapler could be inserted and utilized on the colon adjacent to the fistula. In contrast, the gallbladder was atrophic and hardened. Resection was simple, and accomplished using a 60 mm stapler cartridge. Subsequently, a cholecystectomy was considered; however, the gallbladder was suspected to be nonfunctional. Furthermore, the gallbladder neck was firmly adhered to and embedded within the mesocolon, descending part of the duodenum, and hepatoduodenal ligament. Therefore, any attempt to remove the gallbladder could be associated with a high risk of adjacent organ injury. In addition, there were no residual gallstones observed in the magnetic resonance imagin. As the risk of severe postoperative complications outweighed the benefits or effects of gallbladder removal, we called off a cholecystectomy. To date, no acute or delayed postoperative complications have been reported.

In summary, we encountered a case of colonic gallstone ileus with obstructive colitis secondary to a cholecystocolonic fistula. Obstructive colitis was treated with a transanal ileus tube, followed by spontaneous dislodgement of the stone. Therefore, surgical intervention could be limited to laparoscopic fistula resection with an uneventful postoperative course. Since radical surgical treatment of colonic gallstones and cholecystoenteric fistulas is associated with postoperative morbidity and mortality, this case illustrates the importance of thoroughly considering nonsurgical interventions and surgery for the safe treatment of colonic gallstone ileus.

## CONFLICT OF INTEREST

The authors declare no conflict of interest.

## FUNDING INFORMATION

None.

## ETHICS STATEMENT

All procedures followed have been performed in accordance with the ethical standards laid down Declaration of Helsinki and its later amendments. The ethics committee of this hospital approved the presentation.
